# Supplementary material for: Cathepsin L-dependent positive selection shapes clonal composition and functional fitness of CD4+ T cells
Source: Nat Immunol. 2025 Jun 13;26(7):1127–38. doi: 10.1038/s41590-025-02182-y (PMC12208919; doi:10.1038/s41590-025-02182-y)

nature immunology

Supplementary information https://doi.org/10.1038/s41590-025-02182-y

Cathepsin L-dependent positive selection shapes clonal composition and functional fitness of CD4$^+$ T cells

In the format provided by the authors and unedited

Supplementary Figure 1 - Characterization of *Ctsl*$^{\Delta TEC}$ mice.

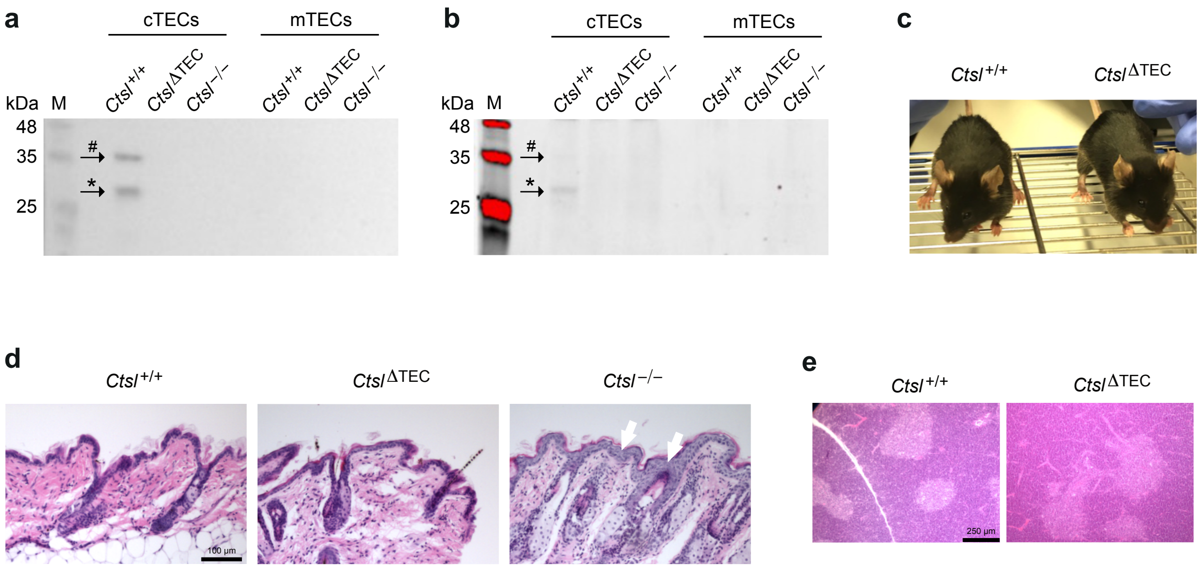

Characterization of *Ctsl*$^{\Delta TEC}$ mice. (a) Cell lysates from cTECs and mTECs sorted from 3-week-old *Ctsl*$^{+/+}$, *Ctsl*$^{\Delta TEC}$ and *Ctsl*$^{-/-}$ mice were separated by SDS-PAGE (3 × 10^4 TECs / lane). Cathepsin L expression was assessed by Western Blot with the polyclonal goat anti-mouse Ctsl antibody AF1515. **(b)** Ctsl activity was assessed by active-site labelling with the BMV109 probe. Arrows indicate pro-Cathepsin L at around 35 kDa (#) and mature Cathepsin L at around 28 kDa (*). **(c)** Morphological phenotype of a *Ctsl*$^{\Delta TEC}$ and a littermate control mouse. **(d)** Hematoxylin and eosin (H&E) staining of paraffin-embedded skin sections from *Ctsl*$^{+/+}$, *Ctsl*$^{\Delta TEC}$ and *Ctsl*$^{-/-}$ mice. White arrows in the *Ctsl*$^{-/-}$ sample denote the typical epithelial hyperplasia seen in these mice, but not in *Ctsl*$^{+/+}$ or *Ctsl*$^{\Delta TEC}$ mice. Representative of n = 3 each. **(e)** H&E staining of paraffin embedded thymus sections from Ctsl$^{+/+}$ and *Ctsl*$^{\Delta TEC}$ mice. Representative of n = 3 each.

Supplementary Figure 2 - Flow cytometric gating strategies

a Gating strategy for cTECs and 'immature' and 'mature' mTECs

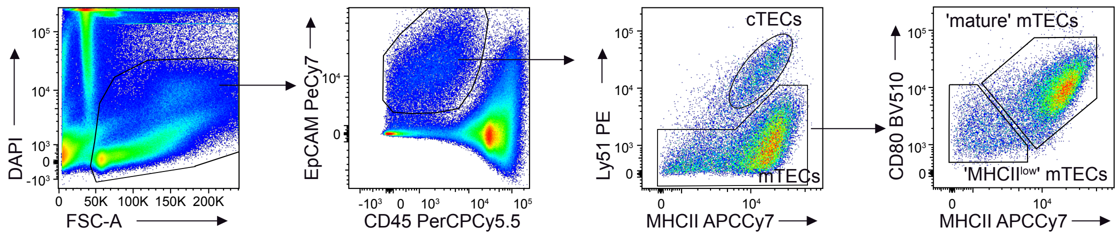

b Gating strategy for Tet+ cells in lymph node or spleen cell suspensions (Magnetically enriched for Tet+ cells)

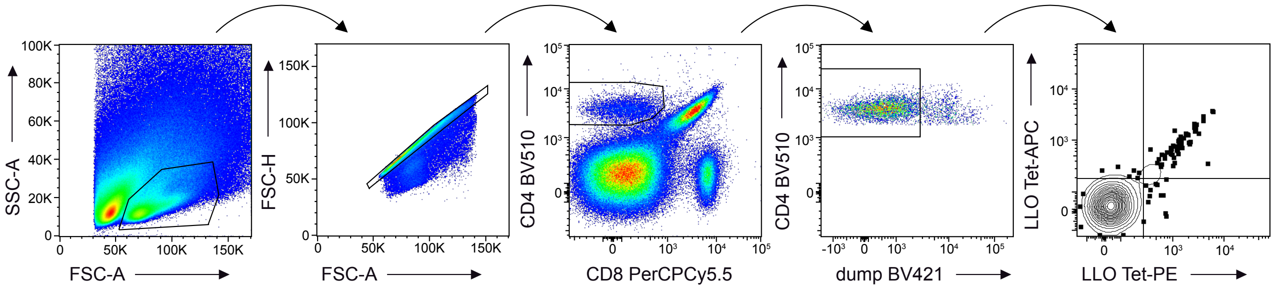

c Gating strategy for Tet+ cells in thymus cell suspensions (Magnetically enriched for Tet+ cells)

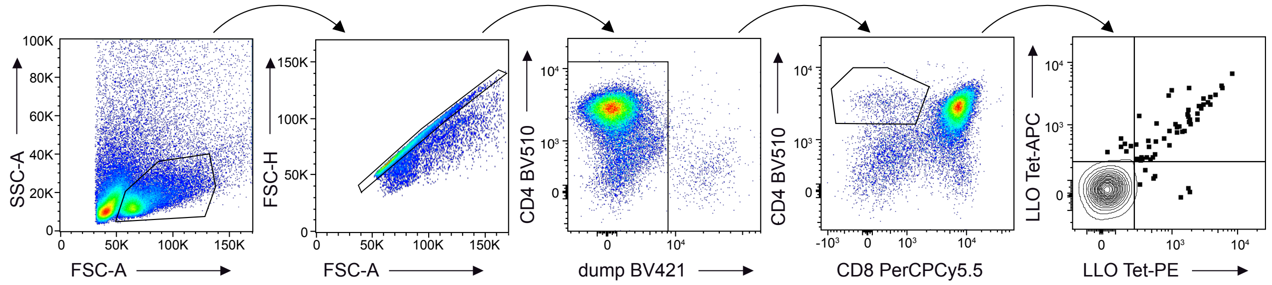

Supplement: Supplementary file 1 — Supplementary Figs. 1 and 2. [file 41590_2025_2182_MOESM1_ESM.pdf]
