# Supplementary material for: Cathepsin L-dependent positive selection shapes clonal composition and functional fitness of CD4+ T cells
Source: Nat Immunol. 2025 Jun 13;26(7):1127–38. doi: 10.1038/s41590-025-02182-y (PMC12208919; doi:10.1038/s41590-025-02182-y)
Supplement: Supplementary file 2 — Reporting Summary [file 41590_2025_2182_MOESM2_ESM.pdf]

Corresponding author(s): Ludger Klein

Last updated by author(s): May 4, 2025

Reporting Summary

Nature Portfolio wishes to improve the reproducibility of the work that we publish. This form provides structure for consistency and transparency in reporting. For further information on Nature Portfolio policies, see our Editorial Policies and the Editorial Policy Checklist.

Statistics

For all statistical analyses, confirm that the following items are present in the figure legend, table legend, main text, or Methods section.

n/a	Confirmed	
☐	☒	The exact sample size (*n*) for each experimental group/condition, given as a discrete number and unit of measurement
☐	☒	A statement on whether measurements were taken from distinct samples or whether the same sample was measured repeatedly
☐	☒	The statistical test(s) used AND whether they are one- or two-sided<br>*Only common tests should be described solely by name; describe more complex techniques in the Methods section.*
☒	☐	A description of all covariates tested
☐	☒	A description of any assumptions or corrections, such as tests of normality and adjustment for multiple comparisons
☐	☒	A full description of the statistical parameters including central tendency (e.g. means) or other basic estimates (e.g. regression coefficient) AND variation (e.g. standard deviation) or associated estimates of uncertainty (e.g. confidence intervals)
☐	☒	For null hypothesis testing, the test statistic (e.g. *F*, *t*, *r*) with confidence intervals, effect sizes, degrees of freedom and *P* value noted<br>*Give P values as exact values whenever suitable.*
☒	☐	For Bayesian analysis, information on the choice of priors and Markov chain Monte Carlo settings
☒	☐	For hierarchical and complex designs, identification of the appropriate level for tests and full reporting of outcomes
☒	☐	Estimates of effect sizes (e.g. Cohen's *d*, Pearson's *r*), indicating how they were calculated

Our web collection on statistics for biologists contains articles on many of the points above.

Software and code

Policy information about availability of computer code

Data collection

Flow cytometry data was acquired using FACSDiva v6.2 (BD Bioscience).
Microscopy images were acquired with LAS X Office v1.4.6 (Leica microsystems).

Data analysis

All analyses are described in the relevant section of the Methods.
Flow cytometry data were analysed with FlowJo v10.9.0.
TCR analyses:
Raw data were de-multiplexed and FASTQ files for each sample were generated using the bcl2fastq software (Illumina).The CLC Genomics Workbench software (v23.0.3) provided by Qiagen was used to generate clonotype reads. TCRα sequencing data were analysed with RStudio (2024.04.2+764), using the R packages iNEXT (Versions 2.0.20 and 3.0.1) and abdiv (Version 0.2.0). An executable capsule containing code and bulk TCRseq data is available at https://doi.org/10.24433/CO.5359169.v1.
RNAseq:
Expression quantification was performed using kallisto (version 0.48) with Ensembl release version 106 for M. musculus. In R/Bioconductor, expression data were collapsed from isoform to gene level for downstream processing. Differential expression was assessed using DESeq2 (version 1.36). Gene Set Enrichment analyses were conducted using fgsea (version 1.22).
Statistical analyses were performed with GraphPad Prism v9.

For manuscripts utilizing custom algorithms or software that are central to the research but not yet described in published literature, software must be made available to editors and reviewers. We strongly encourage code deposition in a community repository (e.g. GitHub). See the Nature Portfolio guidelines for submitting code & software for further information.

Data

Policy information about availability of data

All manuscripts must include a data availability statement. This statement should provide the following information, where applicable:

- Accession codes, unique identifiers, or web links for publicly available datasets
- A description of any restrictions on data availability
- For clinical datasets or third party data, please ensure that the statement adheres to our policy

Sequencing data from this study have been deposited at the GEO and will be publicly available from the date of publication. The accession numbers are GSE269202 for bulk TCRseq data (relating to Figs. 3c-g and 5b-e) and GSE269197 for RNAseq data (relating to Figs. 7 a,b,f). Source data referring to bulk and single-cell TCRseq datasets and bulk RNAseq are provided with this paper.

Research involving human participants, their data, or biological material

Policy information about studies with human participants or human data. See also policy information about sex, gender (identity/presentation), and sexual orientation and race, ethnicity and racism.

Reporting on sex and gender	n.a.
Reporting on race, ethnicity, or other socially relevant groupings	n.a.
Population characteristics	n.a.
Recruitment	n.a.
Ethics oversight	n.a.

Note that full information on the approval of the study protocol must also be provided in the manuscript.

Field-specific reporting

Please select the one below that is the best fit for your research. If you are not sure, read the appropriate sections before making your selection.

☒ Life sciences ☐ Behavioural & social sciences ☐ Ecological, evolutionary & environmental sciences

For a reference copy of the document with all sections, see nature.com/documents/nr-reporting-summary-flat.pdf

Life sciences study design

All studies must disclose on these points even when the disclosure is negative.

Sample size	In a single experiment, at least three biological replicates were used, with the exception of stimulated samples in Fig.6f. As the present study was of exploratory character, no sample size calculation was performed. In general, for these types of experiments, we aim for a group size of 7 each (Allgoewer and Mayer, 2007). When smaller sample sizes were used, the sample size was determined based on previous work or the experience from the first experimental replicate. Moreover, data was collected in repeated independent experiments.
Data exclusions	No data were excluded.
Replication	All major experiments were repeated at least once (as detailed in the figure legends) and replications were successful. The replication number of each experiment is included in the legends.
Randomization	Randomization is not relevant to this study as samples in each experiment were treated uniformly and the same data analysis procedure was applied to all samples of the same experiment.
Blinding	Investigators were not blinded in this study because all results presented are based on quantitative analysis, which is not subject to human biases.

Reporting for specific materials, systems and methods

We require information from authors about some types of materials, experimental systems and methods used in many studies. Here, indicate whether each material, system or method listed is relevant to your study. If you are not sure if a list item applies to your research, read the appropriate section before selecting a response.

Materials & experimental systems

n/a	Involved in the study
☐	☒ Antibodies
☒	☐ Eukaryotic cell lines
☒	☐ Palaeontology and archaeology
☐	☒ Animals and other organisms
☒	☐ Clinical data
☒	☐ Dual use research of concern
☒	☐ Plants

Methods

n/a	Involved in the study
☒	☐ ChIP-seq
☐	☒ Flow cytometry
☒	☐ MRI-based neuroimaging

Antibodies

Antibodies used

Antibody, catalogue #, clone #, supplier

anti-CD28, #BE0015-1, 37.51, Bio X Cell
anti-CD4 BV510, 100559, RM4-5, Biolegend
anti-CD4 biotinylated, 100508, RM4-5, Biolegend
anti-CD8α PeCy7, 100722 , 53-6.7, Biolegend
anti-CD8α PerCPCy5.5, 100734, 53-6.7, Biolegend
anti-CD8α biotinylated, 100704, 53-6.7, Biolegend
anti-CD326/Ep-CAM PeCy7, 118216, G8.8, Biolegend
anti-Ly51 Alexa647, 108312, 6C3, Biolegend
anti-Ly51 PE, 108308, 6C3, Biolegend
anti-CD80 PE, 104708 , 16-10A1, Biolegend
anti-CD5 Alexa647, 100614, 53-7.3, Biolegend
anti-CD5 PE, 100608, 53-7.3, Biolegend
anti-CD5 BV421, 100617, 53-7.3, Biolegend
anti-TCRβ APC, 109212, H57-597, Biolegend
anti-CD69 PE, 104508, H1.2F3, Biolegend
anti-CD69 BV421, 104527, H1.2F3, Biolegend
anti-CD69 PeCy7, 104512, H1.2F3, Biolegend
anti-CD69 BV711, 104537, H1.2F3, Biolegend
anti-H-2Kb FITC, 116505, AF6-88.5, Biolegend
anti-H-2Kb BV786, 742863, AF6-88.5, BD
anti-CD45.1 Alexa647, 110720, A20, Biolegend
anti-CD45.1 FITC, 110706, A20, Biolegend
anti-CD45.1 BV421, 110731, A20, Biolegend
anti-CD45.2 Alexa647, 109818, 104, Biolegend
anti-CD45.2 FITC, 109806, 104, Biolegend
anti-CD45.2 BV421, 109832, 104, Biolegend
anti-CD44 BV421, 103039, IM7, Biolegend
anti-CD44 APC, 103012, IM7, Biolegend
anti-CD25 PeCy7, 102016, PC61, Biolegend
anti-CD62L FITC, 104406, MEL-14, Biolegend
anti-CD62L APCCy7, 104428, MEL-14, Biolegend
anti-TCRvα2 APCCy7, 127818, B20.1, Biolegend
anti-TCRvα2 BV711, 743832, B20.1, BD
anti-TCRvβ5 FITC, 139513, MR9-4, Biolegend
anti-CD127/IL-7Rα PE, 135010, A7R34, Biolegend
anti-CD127/IL-7Rα Alexa488, 135018, A7R34, Biolegend
anti-CCR7 PE, 120106, 4B12, Biolegend
anti-I-A/I-E APCCy7, 107628, M5/114.15.2, Biolegend
anti-CLIP:I-Ab FITC, sc-53946 FITC, 15G4, Santa Cruz Biotechnology
anti-nonCLIP:I-Ab APC, no cat#, BP107.2.2, hybridoma gift from A. Rudensky (Memorial Sloan Kettering Cancer Center)
anti-Ea52-68:I-Ab FITC, Y-Ae, hybridoma gift from B. Kyeswki (German Cancer Research Center)
anti-Nur77 PE, 12-5965-82, 12.14, eBioscience
anti-Bcl2 PE, 633508, BCL/10C4, Biolegend
anti-CD11b PeCy7, 101216, M1/70, Biolegend
anti-CD11b biotinylated, 101204, M1/70, Biolegend
anti-CD11c PeCy7, 117318, N418, Biolegend
anti-CD11c biotinylated, 117304, N418, Biolegend
anti-B220 PeCy7, 103222, RA3-6B2, Biolegend
anti-B220 biotinylated, 103204, RA3-6B2, Biolegend
anti-F4/80 PeCy7, 123114, BM8, Biolegend
anti-F4/80 biotinylated, 123106, BM8, Biolegend
anti-Gr1 biotinylated, 108404, RB6-8C5, Biolegend
goat anti-mouse Cathepsin L polyclonal IgG, AF1515, R&D
mouse anti-goat HRP-conjugated polyclonal IgG, RRID: AB_2339057, 205-035-108, Jackson ImmunoResearch
mouse anti-mouse β-actin,A2228 , AC-15, Sigma

rabbit anti-mouse HRP-conjugated polyclonal IgG, P026002-2, P0260, Agilent Dako

Validation

The antibodies used in this study were used according to the manufacturere's recommendation. Validation was based on the description provided on the manufacturers' homepage Prior to use in experiments, all fluorochrome-labelled antibodies were titrated using a dilutio series on respective antigen positive cells to determine optimal working dilution and performance.
Antibody (clone) Validation:
anti-CD28,https://bioxcell.com/invivomab-anti-mouse-cd28-be0015-1
anti-CD4 (RM4-5) https://www.biolegend.com/de-de/products/brilliant-violet-510-anti-mouse-cd4-antibody-7991
https://www.biolegend.com/de-de/products/biotin-anti-mouse-cd4-antibody-479
anti-CD8α (53-7.3) https://www.biolegend.com/de-de/products/pe-cyanine7-anti-mouse-cd8a-antibody-1906
https://www.biolegend.com/de-de/search-results/percp-cyanine5-5-anti-mouse-cd8a-antibody-4255
https://www.biolegend.com/de-de/products/biotin-anti-mouse-cd8a-antibody-152?GroupID=BLG6765
anti-CD326/Ep-CAM (G8.8) https://www.biolegend.com/de-at/products/pe-cyanine7-anti-mouse-cd326-ep-cam-antibody-5303
anti-Ly51 (6C3) https://www.biolegend.com/de-de/products/alexa-fluor-647-anti-mouse-ly-51-antibody-3310
https://www.biolegend.com/de-de/products/pe-anti-mouse-ly-51-antibody-178
anti-CD80 (16-10A1) https://www.biolegend.com/de-de/products/pe-anti-mouse-cd80-antibody-43?GroupID=BLG1851
anti-CD5 (53-7.3) https://www.biolegend.com/de-de/products/alexa-fluor-647-anti-mouse-cd5-antibody-3199
https://www.biolegend.com/de-de/products/pe-anti-mouse-cd5-antibody-160
https://www.biolegend.com/de-de/products/brilliant-violet-421-anti-mouse-cd5-antibody-8585
anti-TCRβ (H57-597) https://www.biolegend.com/de-de/products/apc-anti-mouse-tcr-beta-chain-antibody-268
anti-CD69 (H1.2F3)https://www.biolegend.com/de-de/products/pe-anti-mouse-cd69-antibody-265
https://www.biolegend.com/de-de/products/brilliant-violet-421-anti-mouse-cd69-antibody-7358
https://www.biolegend.com/de-de/products/pe-cyanine7-anti-mouse-cd69-antibody-3168
https://www.biolegend.com/de-de/products/brilliant-violet-711-anti-mouse-cd69-antibody-12139
anti-H-2Kb (F6-88.5) https://www.biolegend.com/de-de/products/fitc-anti-mouse-h-2kb-antibody-1748
anti-H-2Kb (AF6-88.5) https://www.bdbiosciences.com/en-au/products/reagents/flow-cytometry-reagents/research-reagents/single-color-antibodies-ruo/bv786-mouse-anti-mouse-h-2kb.742863
anti-CD45.1 (A20) https://www.biolegend.com/de-de/products/alexa-fluor-647-anti-mouse-cd45-1-antibody-3104
https://www.biolegend.com/de-de/products/fitc-anti-mouse-cd45-1-antibody-198
https://www.biolegend.com/de-de/products/brilliant-violet-421-anti-mouse-cd45-1-antibody-7255
anti-CD45.2 (104) https://www.biolegend.com/de-de/products/alexa-fluor-647-anti-mouse-cd45-2-antibody-3107
https://www.biolegend.com/de-de/products/fitc-anti-mouse-cd45-2-antibody-6
https://www.biolegend.com/de-de/products/brilliant-violet-421-anti-mouse-cd45-2-antibody-7328
anti-CD44 (IM7) https://www.biolegend.com/de-de/products/brilliant-violet-421-anti-mouse-human-cd44-antibody-7225
https://www.biolegend.com/de-de/products/apc-anti-mouse-human-cd44-antibody-312
anti-CD25 (PC61) https://www.biolegend.com/de-de/products/pe-cyanine7-anti-mouse-cd25-antibody-1929
anti-CD62L (MEL-14) https://www.biolegend.com/de-de/products/fitc-anti-mouse-cd62l-antibody-384
https://www.biolegend.com/de-de/products/apc-cyanine7-anti-mouse-cd62l-antibody-3934
anti-TCRvα2 (B20.1) https://www.biolegend.com/de-de/products/apc-cyanine7-anti-mouse-tcr-valpha2-antibody-7016
https://www.bdbiosciences.com/en-de/products/reagents/flow-cytometry-reagents/research-reagents/single-color-antibodies-ruo/bv711-rat-anti-mouse-v-2-tcr.743832
anti-CD127/IL-7Rα (A7R34) https://www.biolegend.com/de-de/products/pe-anti-mouse-cd127-il-7ralpha-antibody-6190
https://www.biolegend.com/de-de/products/alexa-fluor-488-anti-mouse-cd127-il-7ralpha-antibody-6194
anti-CCR7 (4B12) https://www.biolegend.com/de-de/products/pe-anti-mouse-cd197-ccr7-antibody-2799
anti-I-A/I-E (M5/114.15.2) https://www.biolegend.com/de-de/products/apc-cyanine7-anti-mouse-i-a-i-e-antibody-5966
anti-CLIP:I-Ab (15G4) https://www.scbt.com/p/mhc-class-ii-antibody-15g4
anti-Nur77 (12.14) https://www.thermofisher.com/antibody/product/Nur77-Antibody-clone-12-14-Monoclonal/12-5965-82
anti-Bcl2 (BCL/10C4) https://www.biolegend.com/de-de/products/pe-anti-bcl-2-antibody-6466
anti-CD11b (M1/70) https://www.biolegend.com/de-de/products/pe-cyanine7-anti-mouse-human-cd11b-antibody-1921
https://www.biolegend.com/de-de/products/biotin-anti-mouse-human-cd11b-antibody-346
anti-CD11c (N418) https://www.biolegend.com/de-de/products/pe-cyanine7-anti-mouse-cd11c-antibody-3086
https://www.biolegend.com/de-de/products/biotin-anti-mouse-cd11c-antibody-1814
anti-B220 (RA3-6B2)https://www.biolegend.com/de-de/products/pe-cyanine7-anti-mouse-human-cd45r-b220-antibody-1930
https://www.biolegend.com/de-at/products/biotin-anti-mouse-human-cd45r-b220-antibody-444
anti-F4/80 (BM8) https://www.biolegend.com/de-at/products/pe-cyanine7-anti-mouse-f4-80-antibody-4070
https://www.biolegend.com/de-at/products/biotin-anti-mouse-f4-80-antibody-4066
anti-Gr1 (RB6-8C5)
goat anti-mouse Cathepsin L polyclonal IgG (AF1515)https://www.rndsystems.com/products/mouse-rat-cathepsin-l-antibody_af1515
mouse anti-goat HRP-conjugated polyclonal IgG (205-035-108) https://www.jacksonimmuno.com/catalog/products/205-035-108
mouse anti-mouse β-actin (AC-15) https://www.sigmaaldrich.com/EE/en/product/sigma/a2228
rabbit anti-mouse HRP-conjugated polyclonal IgG (P0260) https://www.agilent.com/en/product/specific-proteins/elisa-kits-accessories/rabbit-anti-mouse-immunoglobulins-hrp-solid-phase-absorbed-2717115

Animals and other research organisms

Policy information about studies involving animals; ARRIVE guidelines recommended for reporting animal research, and Sex and Gender in Research

Laboratory animals

All mice used were on a C57BL/6J background except for F1 B6 x BALB/c in Fig. 1g and were maintained under specific pathogen-free conditions in individually ventilated cages at an ambient temperature of 22°C and 55% humidity with standard light cycle conditions. Ctslfl/fl, Ctsl−/−, TCR-Dep, C2TAkd, TCR-PLP1, TCR-LLO56 and TCR-LLO118, TCR-AND and TCR-AD10, TCR-OT-II, Foxn1-Cre, MHCI−/− (B2m−/−), MHCII−/− (H2-Ab1−/−), Rag1−/−, , Plp1−/−, TCRα−/− and Foxp3GFP reporter (DEREG) mice were previously reported. Lm6, Lm54 and Fixed-β transgenic mice were generated by injection of linearized DNA (encoding the V(D)J regions of the respective TCRs

	identified by single cell TCR sequencing) into pronuclei of C57BL/6 zygotes. All phenotypic analyses were performed in mice of 8–12 weeks of age, unless otherwise indicated.
Wild animals	No wild animals were used in this study
Reporting on sex	Sex-matched controls were used whenever possible. Results presented include both female and male animals, as no sex-specific effects were observed in the tissues analysed.
Field-collected samples	no field collected samples were used in the study.
Ethics oversight	Animal studies and procedures were approved by local authorities (Regierung von Oberbayern) and performed under protocol #Vet_02-22-66.

Note that full information on the approval of the study protocol must also be provided in the manuscript.

Plants

Seed stocks	n.a.
Novel plant genotypes	n.a.
Authentication	n.a.

Flow Cytometry

Plots

Confirm that:

☒ The axis labels state the marker and fluorochrome used (e.g. CD4-FITC).

☒ The axis scales are clearly visible. Include numbers along axes only for bottom left plot of group (a 'group' is an analysis of identical markers).

☒ All plots are contour plots with outliers or pseudocolor plots.

☒ A numerical value for number of cells or percentage (with statistics) is provided.

Methodology

Sample preparation	To obtain single-cell suspensions from thymi, lymph nodes or spleens, organs were dissected, smashed and filtered through a 150µm cell strainer. Splenocytes were additionally subjected to red blood cell lysis by incubation in BD Pharm Lyse™ lysing solution for 5 min at RT.<br>To obtain single-cell suspensions from the bone marrow, femur and tibia bones were dissected, smashed with the use of mortar and pestle and filtered through a 150µm cell strainer. Cells were then subjected to red blood cell lysis by incubation in BD Pharm Lyse™ lysing solution for 1 min at RT.<br>For isolation of TECs, thymi were dissected and cut into pieces. Thymocytes were mechanically released by pipetting up and down and the supernatant containing thymocytes was discarded. The thymus fragments were digested with liberase™ (0.5 U/ml; Roche) and DNase I (10 mg/ml; Roche) at 37°C in three consecutive rounds of 15 min. Cells were filtered though a 80µm cell strainer and washed. Cell pellets were re-suspended in 1 ml of high-density Percoll™ (ρ=1.115; GE Healthcare) and overlaid with 1 ml of low-density Percoll (ρ=1.055), followed by a layer of 1 ml RPMI (Gibco). The gradient was centrifuged at 1350 g for 30 min at 4 °C (w/o brake). The upper interphase, containing the low-density cell fraction, was harvested, washed and subjected to CD45 MACS depletion, using CD45 MicroBeads (Miltenyi Biotech).
Instrument	Sorts were performed on a FACSAriaFusion sorter (BD). Samples were acquired on a FACSCantoII or LSRFortessa (BD).
Software	Analysis of Flow Cytometry data was performed with FlowJo v10.9.0.
Cell population abundance	Post-sort purity was determined by re-acquiring and recording aliquots of the sorted populations. Sorted populations were typically 90-99% pure.

Gating strategy

For thymus/LN samples: 1. FSC-A/SSC-A to exclude cell debris; 2. FSC-A/FSC-H to retain only singlets; 3. CD4/CD8
For tetramer-stained cells: 1. FSC-A/SSC-A to exclude cell debris; 2. FSC-A/FSC-H to retain only singlets; 3. dump/CD3 to exclude tetramer non specific staining; 4. CD4/CD8; 5. TetAPC/TetPE
For TECs: 1. DAPI/CD45 to exclude dead and hematopoietic cells; 2. EpCAM/FSC-A to retain only big cells of the epithelial lineage; 3. Ly51/MHCII to distinguish cTECs and mTECs; 4. CD80/MHCII to distinguish mTEClo and mTEChi
Supplementary Fig S2 shows example gating strategies.

☒ Tick this box to confirm that a figure exemplifying the gating strategy is provided in the Supplementary Information.
